# Fosmidomycin Uptake into *Plasmodium* and *Babesia-*Infected Erythrocytes Is Facilitated by Parasite-Induced New Permeability Pathways

**DOI:** 10.1371/journal.pone.0019334

**Published:** 2011-05-04

**Authors:** Stefan Baumeister, Jochen Wiesner, Armin Reichenberg, Martin Hintz, Sven Bietz, Omar S. Harb, David S. Roos, Maximilian Kordes, Johannes Friesen, Kai Matuschewski, Klaus Lingelbach, Hassan Jomaa, Frank Seeber

**Affiliations:** 1 Parasitologie, Fachbereich Biologie, Philipps-Universität, Marburg, Germany; 2 Institut für Klinische Immunologie und Transfusionsmedizin, Universitätsklinikum Giessen und Marburg GmbH, Giessen, Germany; 3 Department of Biology, University of Pennsylvania, Philadelphia, Pennsylvania, United States of America; 4 Parasitology Unit, Max-Planck-Institute for Infection Biology, Berlin, Germany; 5 Fachgebiet 16 Parasitologie, Robert-Koch-Institut, Berlin, Germany; Université Pierre et Marie Curie, France

## Abstract

**Background:**

Highly charged compounds typically suffer from low membrane permeability and thus are generally regarded as sub-optimal drug candidates. Nonetheless, the highly charged drug fosmidomycin and its more active methyl-derivative FR900098 have proven parasiticidal activity against erythrocytic stages of the malaria parasite *Plasmodium falciparum*. Both compounds target the isoprenoid biosynthesis pathway present in bacteria and plastid-bearing organisms, like apicomplexan parasites. Surprisingly, the compounds are inactive against a range of apicomplexans replicating in nucleated cells, including *Toxoplasma gondii*.

**Methodology/Principal Findings:**

Since non-infected erythrocytes are impermeable for FR90098, we hypothesized that these drugs are taken up only by erythrocytes infected with *Plasmodium*. We provide evidence that radiolabeled FR900098 accumulates in theses cells as a consequence of parasite-induced new properties of the host cell, which coincide with an increased permeability of the erythrocyte membrane. *Babesia divergens,* a related parasite that also infects human erythrocytes and is also known to induce an increase in membrane permeability, displays a similar susceptibility and uptake behavior with regard to the drug. In contrast, *Toxoplasma gondii*-infected cells do apparently not take up the compounds, and the drugs are inactive against the liver stages of *Plasmodium berghei*, a mouse malaria parasite.

**Conclusions/Significance:**

Our findings provide an explanation for the observed differences in activity of fosmidomycin and FR900098 against different Apicomplexa. These results have important implications for future screens aimed at finding new and safe molecular entities active against *P. falciparum* and related parasites. Our data provide further evidence that parasite-induced new permeability pathways may be exploited as routes for drug delivery.

## Introduction

The antibiotic Fosmidomycin (Fos; 3-[formyl(hydroxy)amino]propylphosphonic acid; CID 572) and its derivative FR900098 (FR; 3-[acetyl(hydroxy)amino]propylphosphonic acid; CID 162204) were described previously as inhibitors of DOXP reductoisomerase (Dxr), the second enzyme in the biosynthesis pathway of isoprenoids in *P. falciparum*, inhibiting its *in vitro* and *in vivo* growth at high nanomolar concentrations [1]. Several studies have confirmed the presence of the other individual enzymatic steps in this organism and its essential nature for parasite survival [2], [3]. In combination with the antibiotic drug clindamycin, Fos has already been tested in phase II clinical trials against uncomplicated malaria with good success [4], [5], [6], [7], [8]. Fos has an exceptional safety profile in humans, even when given repeatedly at a dose of 8 g/day [9]. There is an ongoing need for new, safe and affordable anti-malarials, in particular after reports of decreased sensitivity against artemisinin-based monotherapy have appeared in the literature [10].

Isoprenoids are a large and diverse group of natural compounds fulfilling a large number of diverse cellular functions in all biological systems, such as cell signaling processes, protein modifications (prenylation), synthesis of the co-factor ubiquinone and modifications of tRNAs, amongst others [11]. The basic building blocks for all these structures are isopentenyl diphosphate (IPP) and its isomeric form, dimethylallyl diphosphate (DMAPP). Two alternative routes for their synthesis are known: most eubacteria and plants follow the so-called 1-deoxy-D-xylulose-5-phosphate (DOXP) pathway (also called methylerythritol phosphate (MEP) pathway) whereas eukaryotes and archaebacteria mostly use the mevalonate (MEV) pathway [12] (see Figure S1). The two pathways are fundamentally different, starting from different compounds and employing distinct enzymes leading to specific intermediate products. Unlike humans, almost all apicomplexan parasites, including *Plasmodium falciparum*, the causative agent of human malaria, and *Toxoplasma gondii*, causing toxoplasmosis, are now known to synthesize isoprenoids exclusively via the DOXP pathway in the apicoplast, an essential, metabolically active, reduced plastid of endosymbiotic descent found in almost all Apicomplexa [13].

Bioinformatic analyses of the published genome sequences from several Apicomplexa of human and veterinary medical importance (*Plasmodium, T. gondii, Neospora caninum, Theileria* and *Babesia bovis*) have unequivocally shown the presence of all genes of the DOXP pathway in all these organisms [13], [14] (see also Table S1). Notably, the Dxr protein sequences are highly similar among these organisms, and residues known from 3D-structures of bacterial Dxr to be important for Fos binding are well conserved (Figure S2). They can be superimposed onto the respective amino acids in a 3D-model of the *T. gondii* sequence [13]. Given these facts it could be assumed that Fos and FR are also active against those parasites. Surprisingly, however, several reports have shown that Fos does not kill *T. gondii*, *Eimeria tenella* and *T. parva*, even at very high (>100) micromolar concentrations [1], [14], [15], [16].

There are numerous reasons why drugs may be ineffective, but the most obvious one is a failure of its uptake into the infected host cell. We therefore started to investigate whether basic differences exist in Fos uptake between *T. gondii*-infected fibroblasts and cells infected with susceptible parasites, namely *P. falciparum*-infected erythrocytes. The latter induce alterations in the permeability of the red blood cell (RBC) plasma membrane for a variety of different solutes and which are collectively called new permeability pathways (NPP) [17].

Here we provide evidence that these pathways, which appear to be absent in non-infected erythrocytes, greatly facilitate uptake of the respective drugs into the infected cell. Likewise, *Babesia divergens*, another apicomplexan parasite of erythrocytes that is also known to possess NPP-like activities, was found to be susceptible to Fos and its derivative FR. Again, Fos uptake was NPP-mediated. Our results are consistent with the view that parasite-induced changes of the host erythrocyte membrane are pre-requisites for the uptake of these drugs into infected RBC. At the same time they provide a likely explanation for the failure to kill other apicomplexans where NPP appear to be absent or not required.

## Results

### Identification of DOXP reductoisomerase activity in *T. gondii* cell lysates and its inhibition by fosmidoymcin

**Figure 1:**
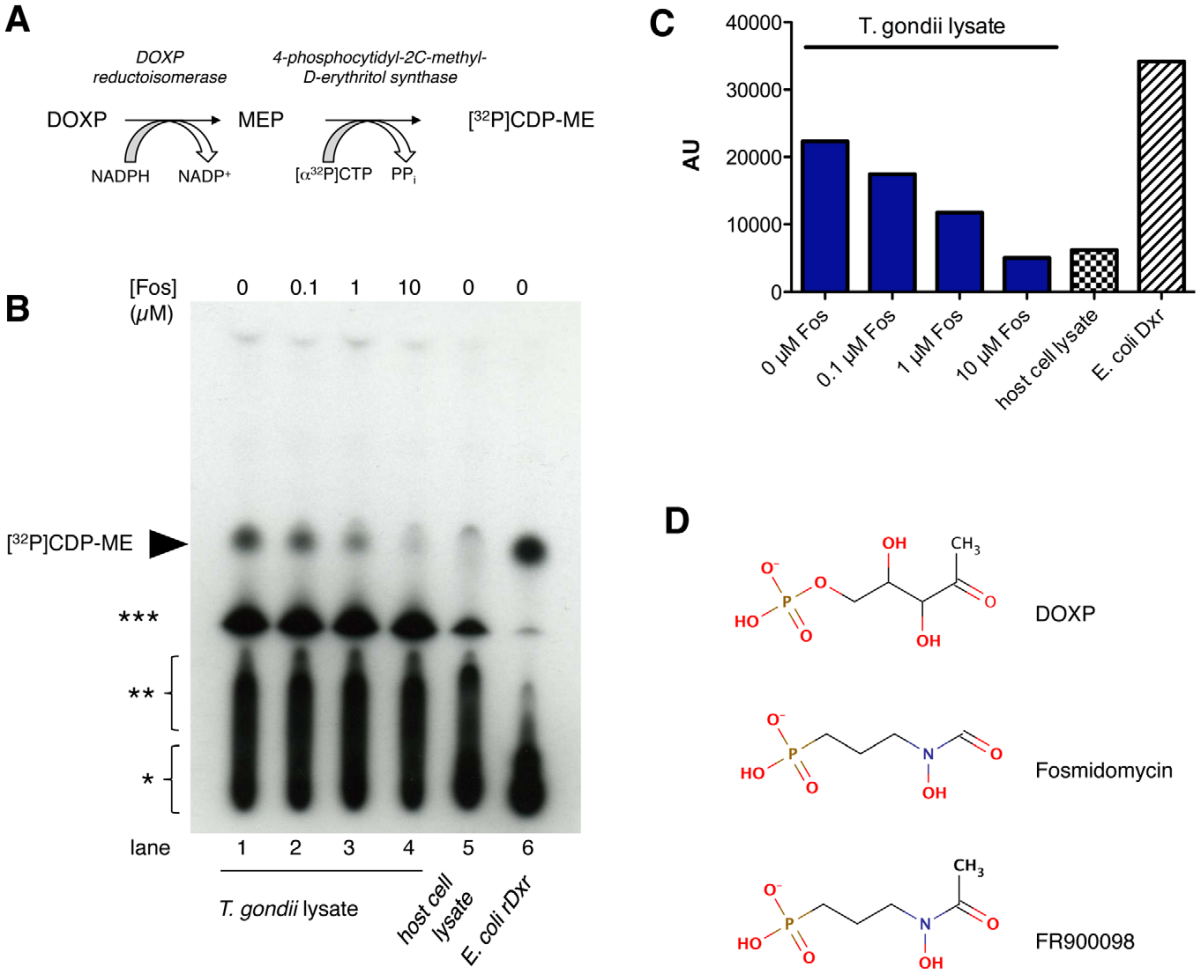
Fosmidomycin-sensitive Dxr activity in *T. gondii.* **A** Principle of the coupled radiometric assay for Dxr activity measurement. MEP formed by Dxr from DOXP and NADPH is converted into [^32^P]CDP-ME in a second reaction using recombinant *E. coli* YgbP (IspD) enzyme and [α-^32^P]CTP. **B** Analysis of the Dxr assay with *T. gondii* lysate in the presence of different concentrations of Fos (lanes 2–4) and without drug (lane 1) by autoradiography of the TLC plate after separation of the reaction products. The appearance of [^32^P]CDP-ME is indicative of Dxr activity (arrowhead). The smear at the bottom is [α-^32^P]CTP (*), the one above (**) is [α-^32^P]CDP whereas the prominent spot below [^32^P]CDP-ME is caused by [α-^32^P]CMP (***). The production of CDP and CMP from CTP is presumably due to the presence of phosphatases in the whole cell lysates, which explains the weaker corresponding signals with the recombinant *E. coli* Dxr protein (lane 6). As controls, either host cell lysate (negative control, lane 5) or 10 pg of purified recombinant *E. coli* Dxr protein (41 U/mg; positive control, lane 6) was used. **C** Densitometric evaluation of the [^32^P]CDP-ME signal from **B**. AU, arbitrary units. **D** Comparison of the structures of the Dxr substrate DOXP with of Fos and FR.

In initial experiments we wished to formally prove that Fos-inhibitable Dxr activity is present in *T. gondii* since functional data on Dxr activity in *T. gondii* have not been reported so far. To evaluate whether native Dxr from *T. gondii* (*Tg*Dxr) is an active enzyme a highly specific and sensitive radiometric assay (see Methods) was performed on lysates of tachyzoite-infected host cells. In this assay, MEP formed from DOXP and NADPH by Dxr is further converted into CDP-ME by recombinant *E. coli* YgbP, the enzyme performing the next step in the DOXP pathway, thereby incorporating the radioactively labeled phosphorus atom from [α-^32^P]CTP (Figure 1A; Figure S1). The results show that (i) significant Dxr acitivity is present only in lysates of cells infected with tachyzoites (Figure 1B, compare lane 1 with lane 5) and (ii) that the activity can be completely inhibited by Fos in a dose-dependent manner (Figure 1B, lanes 2–4; 1C). We conclude that *Tg*Dxr is an active enzyme that can be inhibited by low micromolar concentrations of Fos *in vitro*.

### DOXP reductoisomerase localizes to the apicoplast in *T. gondii* tachyzoites and *P. falciparum* blood stages

**Figure 2:**
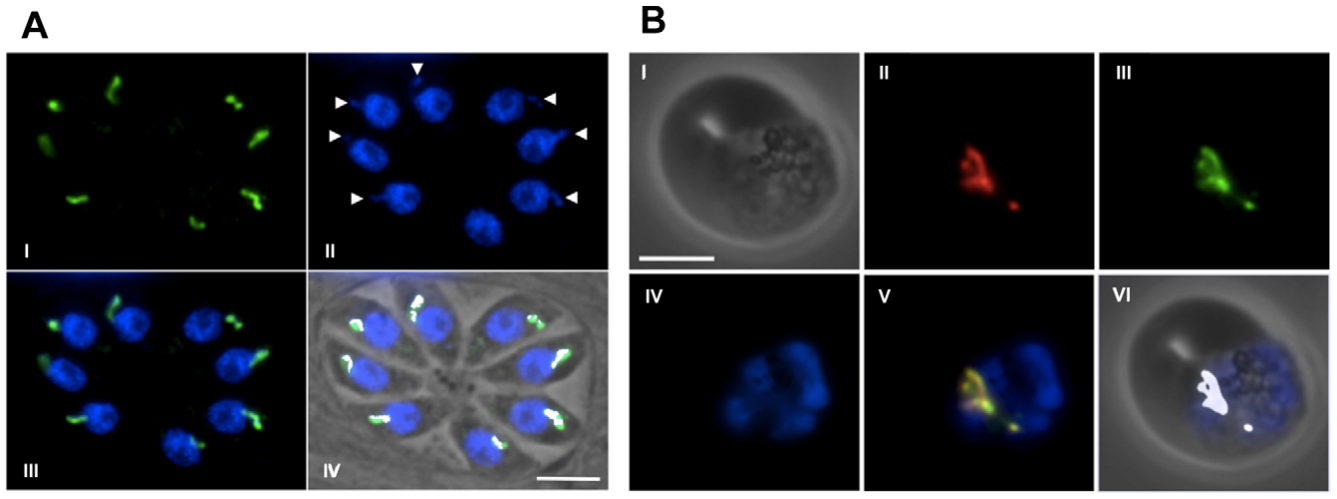
Intracellular localization of Dxr in *T. gondii* tachyzoites and *P. falciparum* blood stages. Antibody staining was performed on fixed parasites with a polyclonal antibody raised against recombinant *P. falciparum* Dxr. **A** Discrete anti-*Pf*Dxr reactivity (green) can be detected in *T. gondii* tachyzoites in the apicoplast (I), co-localizing with organellar DNA (blue; arrowheads in II; overlaid in III). Co-localization is visualized in IV using the ‘Colocalization’ plugin from the ImageJ software suite. White pixels indicate the summed-up overlapping co-localizing signals from III, superimposed onto the phase contrast image of the parasites within the vacuole. **B** Schizont stage of *P. falciparum:* (I) phase contrast of iRBC; (II) anti-*Pf*ACP (red); (III) anti-*Pf*Dxr (green); (IV) DAPI stain (blue); (V) merged images of II-IV (co-localization of anti-*Pf*ACP and anti-*Pf*Dxr (yellow); (VI) merged images of I and of co-localized pixels (using the ‘Colocalization’ plugin applied to II and III). Co-localization of anti-*Pf*ACP and anti-*Pf*Dxr (white). Scale bars = 5 µm.

We next wanted to confirm that Dxr resides in the apicoplast of apicomplexan parasites. Notably, available proteomics data do not provide direct evidence for the expression of Dxr neither in *P. falciparum* nor in *T. gondii* (see Table S1), and neither has *in situ* localization of Dxr in *P. falciparum* or in *T. gondii* been reported so far. Previous targeting experiments had shown that the N-terminal leader peptide of *P. falciparum* Dxr fused to GFP transported this construct to the apicoplast of *T. gondii* tachyzoites [1]. To prove expression of Dxr in the apicoplast, polyclonal antibodies were raised against recombinant *Pf*Dxr (Figure 2 and Figure S11) and antibody staining was performed on intracellular *T. gondii* tachyzoites and *P. falciparum* blood stages. Discrete anti-*Pf*Dxr reactivity can be detected in *T. gondii* tachyzoites (Figure 2A) as well as *P. falciparum* blood stages (schizonts, Figure 2B; for other stages see Figures S3, S4, S5, S6, S7). Using co-localization with the apicoplast-resident acyl carrier protein (*Pf*ACP) in the case of *P. falciparum* and direct staining of the apicoplast DNA for *T. gondii,* these structures were clearly identified as the apicoplast in both organisms, indicating that Dxr is expressed in this organelle. For *P. falciparum* this is in agreement with results showing that most downstream intermediates of Dxr could be detected in all blood stages [2].

### *T. gondii*-infected fibroblasts do not take up FR

Having shown that *T. gondii* possesses Dxr activity that can be inhibited by Fos we directly addressed the possibility that the failure of the drug to kill tachyzoites could be due to its reduced uptake into cells infected with *T. gondii.* To this end we synthesized radiolabeled drug for transport studies. [^14^C]FR is much easier to synthesize than [^14^C]Fos, and FR differs from Fos by a single methyl group (Figure 1D) which neither alters its hydrophilicity nor other physico-chemical properties (Table S2) nor overall shape (Figure S8). FR is also twice as potent in inhibiting *Pf*Dxr than Fos [1].

**Figure 3:**
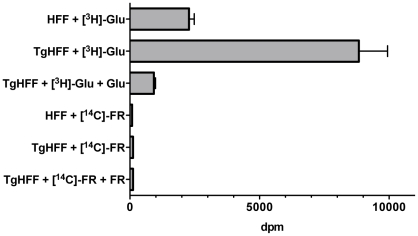
Uptake studies of [^14^C]FR and [^3^H]Glu into human fibroblasts. Duplicates of cells (HFF, non-infected; *Tg*HFF, >50% infected with tachyzoites) were incubated in parallel in phosphate-free “extracellular buffer” (see Material and Methods) for 15 min at 37°C in the presence of either [^14^C]FR or [^3^H]Glu, respectively, of the same specific activity (1.45 µCi/ml, equaling 25 µM drug concentration per assay). Cell-associated radioactivity was determined by scintillation counting of an aliquot of lysed cells and dpm for each compound were determined according to the manufacturer's instructions (shown as dpm±SD). In some assays cells were preincubated for 30 min in 1 mM unlabelled FR or L-Glu to test for uptake specificity (indicated by ‘+FR’ and ‘+Glu’). This is one representative of three similar experiments.

When we followed [^14^C]FR uptake into human foreskin fibroblasts (HFF) for 15 min no significant cell-associated radioactivity was seen for [^14^C]FR (Figure 3). However, [^3^H]-L-glutamate ([^3^H]Glu) that served as control (see below) was readily taken up, as described previously [18]. Heavy infection of cells (>50%) with tachyzoites did not result in higher [^14^C]FR counts, in stark contrast to four-fold increased values for [^3^H]Glu (Figure 3). This is consistent with the observed slight up-regulation of transcripts of the epithelial high affinity glutamate transporter EAAT3 upon *T. gondii* infection [19]. Intracellular accumulation of [^3^H]Glu could be significantly inhibited by pre-incubation with unlabeled L-Glu. Extending the labeling period with [^14^C]FR to 2 hours in similar preliminary experiments did not result in significantly different dpm between these two time points for both, infected and non-infected HFF (unpublished observations). To rule out that rapid efflux of [^14^C]FR via the P-glycoprotein efflux pump was responsible for the observed results assays were performed in the presence of the inhibitor verapamil; however, this did not lead to significantly increased [^14^C]FR counts in those cells (data not shown). Taken together, these experiments indicate that *T. gondii*-infected fibroblasts do not take up FR.

### Uptake of FR into *P. falciparum*-infected erythrocytes depends on functional new permeability pathways (NPP)

**Figure 4:**
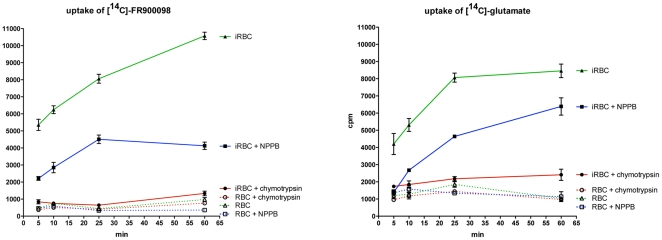
Infection-induced, time-dependent increase of uptake of [^14^C]FR into *P. falciparum* parasitized erythrocytes. Non-infected (RBC) and red blood cells infected with *P. falciparum* (*Pf-*iRBC), respectively, were incubated in RPMI-1640 medium for 60 min containing [^14^C]FR (**A**) or [^14^C]Glu (**B**) of the same specific activity. At the indicated time points aliquots were taken and the radiolabeled tracer in the cellular fraction was quantified (see Material and Methods). Uptake of labeled compounds was also evaluated in the presence of the NPP-inhibitor 5-nitro-2-(3-phenylpropylamino)-benzoic acid (NPPB) at 10 µM, or by pre-treatment of *Pf-*iRBC with 1 mg/ml chymotrypsin for 1 h at 37°C. Similarly treated RBC served as controls. Cpm±SD for three experiments are shown.

Since *P. falciparum* induces permeability changes of the host cell for a variety of different substrates, we next studied [^14^C]FR uptake into *P. falciparum*-infected human red blood cells (*Pf-*iRBC). We observed a time-dependent increase of radioactivity in *Pf-*iRBC (Figure 4A). Strikingly, non-infected cells (RBC) showed only minor amounts of cell-associated counts. This largely increased uptake behavior into iRBC is reminiscent of compound entry via the so-called new permeability pathways (NPP). This is illustrated by uptake of the known NPP substrate [^14^C]L-Glu (Figure 4B). To test whether [^14^C]FR entry is indeed via the NPP, we inhibited these pathways pharmacologically with 50 µM 5-nitro-2-(3-phenylpropylamino)-benzoic acid (NPPB), a well-known NPP inhibitor (Figure 4 A,B). Consistent with its entry via the NPP, FR uptake was considerably inhibited (>50%) by NPPB (Figure 4A, B; iRBC+NPPB). Similar results were obtained with another NPP inhibitor, furosemide (data not shown). Both compounds decreased [^14^C]FR uptake dose-dependently (Figure S9).

It has been reported previously that limited protease treatment of *Pf-*iRBC with chymotrypsin almost completely abrogates NPP activity [20]. Accordingly, chymotrypsinization of *Pf-*iRBC resulted in a drastic decrease of FR uptake to almost background levels (Figure 4A, iRBC+chymptrypsin), as did the known NPP-substrate L-glutamate (Figure 4B). Together, these results clearly implicate a role of NPP in FR uptake into *P. falciparum*-infected erythrocytes.

### FR entry via NPP is also observed in *Babesia*-infected erythrocytes

**Figure 5:**
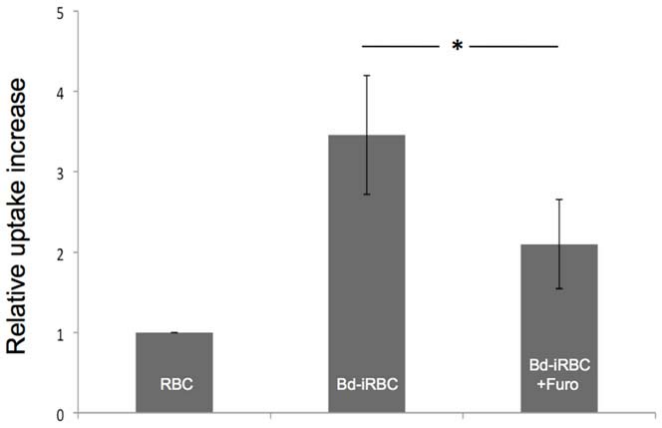
Increased uptake of [^14^C]FR into erythrocytes infected with *Babesia divergens* (*Bd-*iRBC). *Bd-*iRBC were incubated in the presence of [^14^C]FR at 37°C for 20 min before the cells were harvested and the amount of intracellular [^14^C]FR determined. Non-infected RBC and *Bd-*iRBC treated with 100 µM furosemide (Furo) served as controls. Shown are the mean values of three independent experiments (±SD). The data represent the amount of intracellular [^14^C]FR in relation to the amount quantified for RBC (control). * indicates a p-value of 0.011.

Some apicomplexan parasites of the genus *Babesia* also infect mammalian erythrocytes and are considered emerging human pathogens [21]. Recent data provided evidence for permeability changes in *Babesia divergens*-infected human RBC (*Bd-*iRBC) [22]. To test if FR uptake via NPP-like mechanisms is a general feature of Apicomplexa-infected RBC we used *B. divergens* previously adapted to *in vitro* culture in human RBC to measure [^14^C]FR uptake. The results show that *Bd-*iRBC, like *Pf-*iRBC, have significantly higher cell-associated [^14^C]FR counts than non-infected cells (Figure 5). Uptake of [^14^C]FR is not as prominent as in *P. falciparum*-infected erythrocytes, most likely because of a lower trophozoite stage parasitemia, which is due to the current lack of methods for stage-specific enrichment of *Babesia*-infected RBC (average parasitemia of 20% trophozoite stage *Bd-*iRBC, compared to 80% trophozoite stage *Pf-*iRBC). In good agreement with a direct role of NPP-like mechanisms in FR uptake, inhibition by furosemide leads to a statistically significant decreased [^14^C]FR uptake (P = 0.011 by paired t-test).

Having shown that FR also enters *Bd-*iRBC it was of interest whether the drug would also inhibit the intracellular growth of *B. divergens* at pharmacologically relevant concentrations. A sharp reduction in parasite growth (10% of untreated control after 72 h) could already be seen with 10 µM FR (Table 1). Our results are in very good agreement with recently published data showing that Fos inhibits the intraerythrocytic growth of two other *Babesia* species (*B. bovis* and *B. bigemina*) in bovine RBC at such low concentrations (reported IC_50_<5 µM; [23].

**Table 1:** *In vitro* effects of 72 h treatment with FR900098 on growth of *B. divergens.*

FR900098 [µM]	% parasitemia (cells infected/100 cells±SD)	% of untreated
0	43.3±3.4	100
1	36.2±4.4	83.6
10	4.5±1.8	10.4
100	1.8±0.2	4.2

### Failure of FR to inhibit growth of liver stage *P. berghei* parasites *in vitro* and *in vivo*

**Figure 6:**
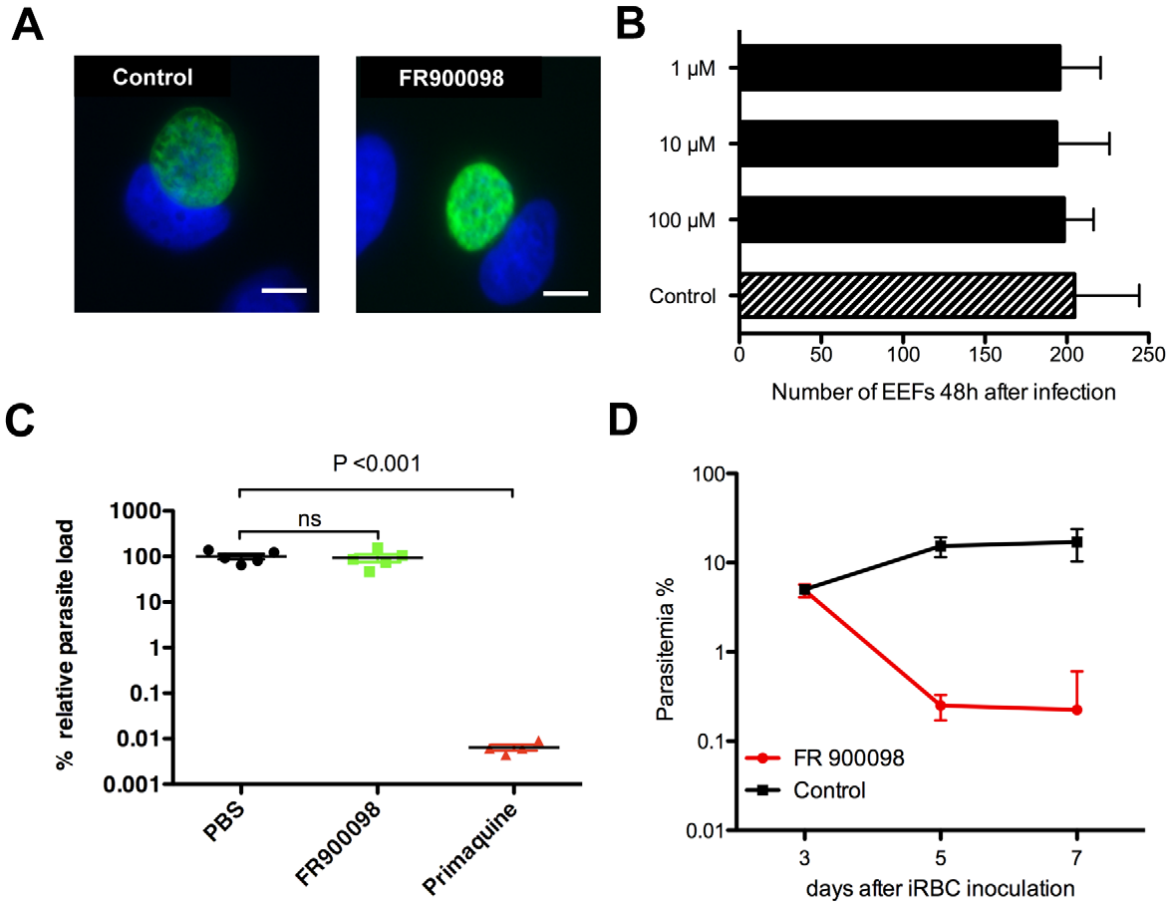
Effect of FR900098 on *P. berghei* liver stage parasites (exoerythrocytic forms, EEF) *in vitro* and *in vivo*. Human hepatoma cells infected with 10,000 GFP-expressing sporozoites and treated with 100 µM, 10 µM and 1 µM FR, respectively, were fixed 48 h p.i. Parasites were visualized with a monoclonal antibody directed against HSP70 and Alexa488-labelled secondary antibody (*), and Hoechst 33342 to stain the nuclei. **A** Immunofluorescence images of untreated and 100 µM-treated EEFs, respectively, showing no changes in morphological appearance upon FR treatment. Bar represents 10 µm. **B** Quantitative assessment of drug effects on parasite growth at the three different FR concentrations. Given are numbers of EEFs/well (mean value±SEM). **C** Quantitation of *in vivo* effect of FR treatment by real time PCR to assess parasite load in livers of infected C57BL/6 mice either untreated, treated with FR or, as a positive control, treated with primaquine. Parasite load on the y-axis is given as the percentage of the mean parasite load (±SEM) in PBS-treated control mice. **D** Effect of the batch of FR used in A-C on blood stage *P. berghei* in infected C57BL/6 mice. Parasitemia was determined by means of Giemsa-stained thin blood smears at the indicated time points after infection.

To our knowledge, the growth inhibiting activity of Fos and FR for Plasmodia has only been studied on blood stage parasites. Based on published microarray data of the liver stages of the rodent parasite *P. yoelii* many of the genes for the DOXP pathway are assumed to be expressed at least as strongly in liver stage as in blood stage parasites ([24]; Figure S10). We therefore tested whether the stages of *P. berghei* multiplying in nucleated liver cells (exo-erythrocytic forms; EEFs) are sensitive to increasing FR concentrations (Figure 6). Human hepatoma cells infected with GFP-expressing *P. berghei* sporozoites were incubated with 1, 10 and 100 µM FR, respectively, for 48 h and parasite replication was recorded. These drug concentrations are between 2 to 200-fold above the IC_50_ for *P. falciparum* blood stages [1]. In no case were we able to detect differences in EEF growth (Figure 6A, B).

We then infected groups of susceptible C57BL/6 mice with 10,000 *P. berghei* sporozoites each and subsequently treated them intraperitoneally with four high doses of 250 mg/kg FR at 12 h intervals. This high dose regimen is expected to result in sustained high drug levels in the liver [25], [26]. However, no significant difference (P>0.05; unpaired student's t-test) of parasite load, as quantified by real-time PCR of *P. berghei* 18S RNA transcripts in the liver of these animals, could be observed after 42 h when compared to those of uninfected control animals (Figure 6C). In contrast, a control group that had received 60 mg/kg primaquine at 0 h und 24 h post-infection showed a vast decline in parasite load, illustrating the susceptibility of this parasite strain to *in vivo* drug treatment. To further prove that the batch of FR used in the above experiments was able to kill susceptible parasites, growth inhibition of blood stages was monitored in a group of mice that had received doses of 75 mg/kg FR every 8–12 hours for 5 days starting at day 3 after iRBC inoculation. Parasitemia in treated animals was 0.22%±0.38% 7 days after infection, whereas that of untreated controls was 17.1%±6.8% (Figure 6D). Taken together these results indicate that susceptibility of *Plasmodium* to FR treatment can only be observed when the parasites reside in erythrocytes but not when multiplying in nucleated liver cells.

## Discussion

We provide evidence that only erythrocytes with an increased plasma membrane permeability for a variety of solutes upon infection with *P. falciparum* or *B. divergens* parasites (so-called new permeability pathways, NPP [17]) are permissive for uptake of the highly charged drugs Fos and FR. In striking contrast, those parasites that do not reside in erythrocytes and do not show this property, such as *T. gondii* or the liver stages of *P. berghei*, are obviously secluded from the drug's action since these compounds seem unable to traverse the plasma membrane of the respective host cells.

The high hydrophilicity of the compounds tested presumably prevents their passage through the host cell plasma membrane in the absence of a suitable transporter or altered membrane permeabilities. This situation resembles that in a number of organisms like the cyanobacterium *Synechocystis* sp. PCC6803 and in Mycobacteria where Fos is known to inhibit the respective bacterial Dxr enzymes but shows no activity against the intact organisms [27], [28], [29]. A recent study provided evidence that Fos is unable to enter Mycobacteria, presumably because they lack the gene for a glycerol-3-P transporter (GlpT) [30], which is also absent in the genome of *Synechocystis* sp. PCC6803 (unpublished observation). In *E. coli* this transporter has been shown to be responsible for Fos uptake [31], and introduction of this gene into *Brucella abortus* is both required and sufficient to make these bacteria Fos-susceptible [32]. Lack of uptake is presumably also the reason why a distant relative to Apicomplexa, the dinoflagellate *Perkinsus marinus*, is insensitive to high concentrations of Fos [33], despite the presence of the whole DOXP pathway in its genome [34] (Figure S2).

From more than 40 compounds known to act on apicoplast targets, Fos and FR are by far the most hydrophilic compounds at physiological pH. Both drugs bear significant structural resemblance to DOXP (see Figure 1D) but the latter is not known to be involved in any human metabolic pathway as a precursor or product [2]. Moreover, in the Human Metabolome Database there is no indication for its presence in body fluids (http://www.hmdb.ca/metabolites/HMDB12173). It is therefore likely that no specific transporter for these compounds exist in the host cell plasma membrane. Only erythrocytes that, upon infection with either *Plasmodium* or *Babesia*, alter their membrane permeability in turn become permissive for Fos or FR uptake. To our knowledge NPP-like permeability changes have not been reported to date for any nucleated cells infected by other Apicomplexa. Of note, *P. berghei* sporozoites modulate the volume-regulated anion channel activity of its target cells upon infection [35]. However, the altered activity is a mere response of the host cell to the increased cell volume and not a prerequisite for parasite survival [35].

Although our data provide clear evidence that Fos or FR uptake into infected erythrocytes requires functional NPP, at present we do not know any molecular entity that is involved in this initial entry process. The existence and importance of NPP in *Plasmodium* (and by comparable mechanisms also in *Babesia*)-infected cells for the acquisition of many essential nutrients is largely undisputed, however, the exact way this is accomplished and the molecules being involved is much less so (for recent discussions see [36], [37]). The recognition of NPP-specific anti-parasitic compounds in this and other studies [38], [39] may aid in the identification of the molecular make-up of the NPP, for instance by exploiting differential screens of *Plasmodium* mutants for Fos resistance.

Fos (in combination with clindamycin) is the first anti-malarial drug that successfully completed phase II trials [8] for which specific cell entry via the NPP pathway is shown. Our findings reinforce the previously suggested concept of exploiting this phenotype for targeting drugs into iRBC [40], [41], [42]. In some parasite lines the development of resistance to antimalarials such as blasticidin and leupeptin has been found to be associated with alterations of a so-called plasmodial surface anion channel (PSAC) [43], [44], indicating the existence of a pathway that involves a parasite-encoded transporter, which may undergo mutations under selective pressure. However, since it is likely that more than a single mechanism constitute what is collectively called NPP [37], it is also possible that in some instances the actual transporter is an activatable host cell protein. In such cases, the chances for resistance development at the point of drug transport should be minimal since there is no selective pressure on the respective host genes. So far experimentally induced resistance against Fos has only been reported to be caused by amplification of the Dxr gene locus [45].

NPP are generally described as transporting a broad range of substrates, with a preference for anions and electroneutral compounds over cations, and with the rate of permeation influenced by size and hydrophobicity of the solute in a number of cases [17]. However, simple correlations between size, charge or hydrophobicity and permeation rates are not apparent, and a combination of different properties could play a role in other cases [46]. Fos and FR are small but very hydrophilic at physiological pH, whereas two other anti-plasmodial compounds (pentamidine; T16) taken up via the same route [38], [39] are larger and much more lipophilic (Table S2). Therefore, predictions of inhibitors that enter iRBC via NPP seem unreliable and instead rigorous experimental testing is required in each case.

The concept that hydrophilic, charged molecules could be potent drugs if their structures resemble natural metabolites for which specific transport routes exist, has been recently emphasized [47]. Extending this idea to *Plasmodium*-infected cells we propose that future screens aimed at finding such new anti-plasmodial molecules should include more small hydrophilic metabolite-like compounds specific to ‘parasite-only’ pathways (e.g. from the apicoplast; [13]) in their chemical libraries (in contrast to the current situation; see [47], [48] for a general discussion). This strategy would increase the chances of developing novel drug candidates with a high, built-in safety profile, provided that they can enter iRBC only via NPP. The fact that the target of Fos and FR, Dxr, is absent from the human host adds an additional safety level to its use. Interestingly, in a recent high-throughput screen of nearly 2 million compounds against blood-stage *P. falciparum*, among 13,500 active hits were also two purine analog phosphonates with physico-chemical properties comparable to Fos and FR [49]: CHEBI 390944 with an estimated IC_50_ of 470 nM, and CHEBI 641822 with an estimated IC_50_ of 340 nM ([49]; Figure S8 and Table S2). It would be interesting to see whether the presence of the purine moieties in these anti-plasmodial phosphonates make them substrates for specific transporters or if they can enter iRBC only via NPP. The latter would further advocate an appealing option [50], namely synthesizing chimeric drugs based on active phosphonates like Fos/FR that are still taken up via the NPP, and subsequently cleaved inside the cell to liberate two or more inhibitory entities.

Our results provide a plausible explanation for the observed differences in killing activity of Fos and FR against different apicomplexan parasites. At present it is not known if any of the other three membranes both compounds have to pass to reach their target Dxr in the apicoplast are also impermeable for these drugs in *T. gondii,* the liver stages of *Plasmodium*, or any of the other apicomplexans that are not killed by Fos or FR. This aspect is of general cell biological and biochemical interest and deserves further studies. However, with regard to Fos' and FR's intended application as drugs these potential barriers are of secondary importance since the host cell plasma membrane is the first and thus determining barrier that has to be overcome by any pharmacologically active compound. Failure to do so eliminates Fos and FR in their current formulation as potential drugs in those host-parasite systems.

Nevertheless, in pathogens that fail to take up Fos or FR the DOXP pathway remains a prime drug target. Hope to improve drug delivery to the pathogen comes from recent studies showing that lipophilic Dxr inhibitors structurally unrelated to Fos and FR can be developed [51]. The hydrophilicity problem of phosphonates is well known and might be overcome by the development of phosphonate esters [52], although cleavage of the ester linkage by cellular esterases in the host cytosol might generate again a charged Fos or FR molecule impermeable for the parasite plasma membrane. Importantly, specific inhibitors for other enzymes of the DOXP pathway in bacteria have been described recently [53], [54].

In conclusion, our results show that the parasite- and life cycle-specific action of anti-infectives may be explained by differential uptake into the infected host cell, which has important implications for future screens aimed at finding safe, affordable and potent molecular entities active against *P. falciparum* and other related Apicomplexa.

## Materials and Methods

### Ethics Statement

All animal work was conducted in accordance with the current German Protection of Animals Act (BGBl. I S. 1207), which implements the directive 86/609/EEC from the European Union and the European Convention for the protection of vertebrate animals used for experimental and other scientific purposes. The protocol was approved by the ethics committee of the Max-Planck-Institute for Infection Biology and the Berlin state authorities (LAGeSo Reg# G0469/09).

### Chemicals

All chemicals were from Sigma-Aldrich (except where noted). Fosmidomycin and FR900098 were synthesized as previously described [55], [56]. [^14^C]-FR900098 was synthesized from [1-^14^C]acetyl chloride as radioactive precursor. The final preparation was characterized by a specific activity of 0.26 mCi/mg (58 mCi/mmol) and a radiochemical purity of 98.8%. [α-^32^P]CTP and [1-^14^C]acetyl chloride was from GE Healthcare, [^14^C]- and [^3^H]-labeled L-glutamic acid were obtained from Hartmann Analytic GmbH, Braunschweig, Germany.

### Cell culture

#### *P. falciparum*

The *in vitro* culturing and synchronization of *P. falciparum* (isolate FCBR) was carried out by standard protocols [57], [58]. Human erythrocytes and plasma were purchased from Blood Bank Universitätsklinikum Gieβen und Marburg GmbH, Germany. Parasites were cultured in human erythrocytes (blood group A^+^) at 37°C and a hematocrit of 2%. The culture medium was RPMI1640 (PAA, Germany) supplemented with 10% human plasma (A^+^; heat inactivated at 56°C for 30 min). Trophozoite-infected erythrocytes were enriched to a parasitemia of >80–90% by plasmagel floatation [59].

#### *T. gondii*

Propagation of *T. gondii* strain RHβ1 in immortalized human foreskin fibroblasts (HFF; hTERT-BJ1; Clontech) was performed as described previously [60].

#### *B. divergens*

Blood stage parasites of *B. divergens* (isolate Rouen 1987; [61] were cultured *in vitro* in human A^+^ erythrocytes (Blood Bank Marburg, Germany) using RPMI1640 supplemented with 10% human serum (A^+^; Blood Bank Marburg, Germany) and 0.2 mM hypoxanthine (c.c. pro, Germany) at 37°C and a hematocrit of 5%. Flasks were flushed with a gas mixture of 90% N_2_, 5% O_2_ and 5% CO_2_. The medium was changed daily and cultures were diluted to 1% once they reached a parasitemia of 20–30%.

### Production of anti-*Pf*Dxr antibodies

For the immunization of rabbits recombinant Dxr of *P. falciparum* (*Pf*Dxr) was produced by an optimized version of a previous protocol [1]. A synthetic gene for *Pf*Dxr adapted to the preferred codon usage of *E. coli* was inserted into the pQE31 expression vector (Quiagen, Hilden, Germany) providing an amino-terminal His_6_ tag. *E. coli* XL1-blue pREP cells were transformed with this construct and grown in Terrific Broth medium at 37°C until an OD_600_ of 1. After induction by the addition of 1 mM IPTG the culture was continued for 15 h at 30°C reaching an OD_600_ of 5 to 9. The cells were harvested by centrifugation, resuspended in a 10-fold volume of IMAC buffer (100 mM NaCl, 30 mM Tris-HCl, 2 mM 2-mercaptoethanol, 14% glycerol, pH 8.0) and disintegrated by ultrasonic treatment (3 times 5 min with 5 min pause, VS70T sonotrode, 30% pulse, 60% amplitude). Insoluble material was removed by centrifugation (75,000 g, 25 min, 4°C) and filtration through a 0.22 µm filter. The soluble fraction was loaded on an immobilized cobalt column (Talon Superflow, Clontech), which was eluted with a two-step gradient of 50 and 150 mM imidazole in IMAC buffer. *Pf*Dxr was obtained in the 150 mM imidazole fraction with a purity of ca. 90% as judged by SDS-PAGE. The specific activity determined in a photometric Dxr enzyme assay was 2 U/mg [62]. The protein was concentrated by ultrafiltration and stored at −70°C. A typical purification procedure starting with 8 flasks each containing 350 ml bacteria culture resulted in ca. 1 mg *Pf*Dxr. The relatively low yield was due to the fact that most of the protein was produced as inclusion bodies.

The purified enzyme was used for custom immunization (Eurogentec, Seraing, Belgium) of 2 rabbits applying a protocol including an initial injection followed by 3 booster shots. For each injection 1.6 mg *Pf*Dxr were used. The sera were tested for reactivity against *Pf*Dxr by Western blot. Only one of the two rabbits developed a sufficiently high specific antibody titer (see Figure S11).

### Immunofluorescence assays of *T. gondii* and *P. falciparum*

For indirect immunofluorescence assays, *T. gondii*-infected HFF cells grown on 22 mm glass cover slips were processed exactly as previously described [63]). Briefly, cells were fixed in 4% paraformaldehyde, permeabilized in 0.25% Triton-X 100, and blocked in 3% BSA fraction V (Fisher). *P. falciparum*-infected RBCs were processed exactly as described by Tonkin et al. [64]. All primary and secondary antibody incubations were carried out at room temperature for 1 h each in blocking solution, followed by three 10 min washes in 0.1% Triton-X 100. Apicoplast and nuclear DNA were stained with 2.8 µM 4′,6-diamidino-2-phenylindole (DAPI, Invitrogen) for 5 min (in PBS) right after the secondary antibody step, and mounted on glass slides using Fluoromount-G (Southern Biotechnology Associates, Inc). Samples were examined on a Leica DM IRBE, 100 W Hg-vapor lamp and an Orca-ER digital camera (Hamamatsu). Images were captured and analyzed using Openlab software (Improvision). For co-localization analysis the ‘co-localization’ plugin of the ImageJ program (Rasband, W.S., ImageJ, U. S. National Institutes of Health, Bethesda, Maryland, USA, http://rsb.info.nih.gov/ij/, 1997–2009) was used.

### Uptake assays

#### Human erythrocytes*/P. falciparum*

Non-infected and infected erythrocytes (2×10^8^ cells/ml with a parasitemia of ∼80%) at the trophozoite stage were incubated at 37°C in RPMI1640 medium without human serum in the presence of [^14^C]FR or [^14^C]Glu, respectively, of the same specific activity (1 µCi/ml).

At the indicated time points, aliquots of 100 µl corresponding to 10^7^ cells were removed and spun through a layer of 200 µl dibutylphtalate. After freezing the sample in liquid nitrogen the bottom of the tube containing the cell pellet was cut, cells were lysed in scintillation cocktail and subsequently radioactivity of the samples was determined in a Beckman Coulter MR4000 scintillation counter. Inhibitor studies using NPPB, furosemide and chymotrypsin were done as described previously [20], with the exception that [^14^C]FR or [^14^C]Glu were added and the cultures were then processed as described above.

#### Human erythrocytes*/B. divergens*

To determine the influx of [^14^C]FR into *B. divergens* infected erythrocytes 10^8^ infected cells with a parasitemia of 15–20% were washed three times in Dulbecco's phosphate buffered saline (DPBS) and subsequently incubated in DPBS for 30 minutes at 37°C, to reach a point of zero membrane transport. Thereafter, cells were incubated in 1 ml DPBS containing 1.7 µCi/ml at 37°C for 20 min. The incubation was carried out in the presence or absence of 100 µM furosemide. To separate the cells from the radioactive medium, aliquots of 100 µl were centrifuged through a cushion of 600 µl dibutylphtalate at 13,000 rpm for 2 min. The resulting pellet was carefully harvested and lysed in 2 ml scintillation cocktail (Roth) and analyzed in a scintillation counter (Beckman LS 6000SC).

#### Human fibroblasts*/T. gondii*

HFF (hTERT-BJ1) cells were cultured in 6-well plates (Corning) to confluency (1×10^6^ cells) in DMEM incl. 10% fetal bovine serum and penicillin/streptomycin. Where appropriate they were infected in duplicate with *T. gondii* tachyzoites (RHβ1) resulting in an infection rate >50% of cells 24 h post infection. Depending on the assay the cells were pre-treated with 1 mM unlabelled FR or Glu for 30 min before they were incubated with either [^14^C]FR or [^3^H]Glu, respectively, of the same specific activity (1.45 µCi/ml, equaling 25 µM drug concentration per assay) for 15 min at 37°C. To rule out a possible competition between phosphate present in DMEM and the phosphonate FR for uptake into cells, labeling was performed in phosphate-free “extracellular buffer” which mimics the extracellular milieu [65]. Labelling was terminated by 3 washes of the monolayers with ice-cold phosphate buffer (50 mM sodium phosphate pH 8.2) containing 1 mM unlabelled FR and Glu each. At this stage microscopic examination of the plates showed that all monolayers were still intact and evenly infected. Cell lysis was performed by incubating the plates on a shaker with 500 µl lysis solution (0.1 M NaOH, 0.1% SDS) for 1 h at 4°C, before an aliquot (200 µl) was counted in a PerkinElmer MicroBeta Trilux scintillation counter. Dpm were determined according to the manufacturer's instructions.

### Coupled radiometric Dxr activity assay

The assay was basically performed as described previously [66]. For preparation of the cell lysate, 10 µl of packed purified *T. gondii* tachyzoites (ca. 10^7^ cells) were suspended in 600 µl assay buffer (100 mM Tris-HCl, 20 mM NaF, 10 mM MgCl_2_, 1 mM MnCl_2_, pH 8.0) supplemented with a protease inhibitor cocktail (4 mM Pefabloc SC, 2 µg/ml aprotinin, 2 µg/ml leupeptin, 2 µg/ml pepstatin A, 2 µg/ml antipain), disintegrated by sonication for 2 min (Sonoplus HD 70, Bandelin, Berlin, Germany; 30% pulse, 70% amplitude) and centrifuged (22,000 *g*, 20 min, 1°C). For the inhibition test, 45 µl aliquots of the lysate were combined with 5 µl of a 20-fold concentrated Fos solution in water and pre-incubated for 5 min on ice. Then, the activity test was started by combining 10 µl of these aliquots with 10 µl of reaction mixture consisting of 8 mM DOXP, 8 mM NADPH, 0.5 mCi/ml [α-^32^P]CTP (400 Ci/mmol) and 0.1 mg/ml *E. coli* YgbP (IspD) in assay buffer. After incubation at 37°C for 5 min, 0.7 µl samples were spotted onto 10×20 cm silica gel 60 HPTLC plates (Merck), which were developed longitudinally for 320 min with a mixture of *n*-propanol/ethyl acetate/H_2_O (6∶1∶3, v/v). For autoradiography, the plates were exposed for 3 h to a Kodak X-Omat AR film.

### *P. berghei* blood stage growth inhibition assay in mice

10 C57BL/6 mice were intravenously infected with 30,000 *P. berghei* ANKA infected red blood cells from a NMRI donor mouse. At day three post-infection (p.i.), parasitemia was determined by means of Giemsa-stained thin blood smears at different time intervals. The baseline parasitemia before addition of the first dose of 75 mg/kg FR900098 was 4.9%±0.66% (mean±standard deviation). 5 mice were treated with 75 mg/kg FR900098 dissolved in PBS, which was given intraperitoneally every 8–12 hours for 5 days. Control mice received PBS only.

### *In vitro* effects of FR900098 on exoerythrocytic forms of *P. berghei*

Labtek slides with 30,000 HuH7 human hepatoma cells [67] were infected with 10,000 sporozoites per well (cl. 507, constitutively expressing GFP; [68]) and treated in triplicate with 100 µM, 10 µM and 1 µM FR900098, respectively. 48 hours after infection cells were fixed with cold methanol. Parasites were visualized with a mouse monoclonal antibody to HSP70, followed by a goat Alexa488-labelled antibody directed against mouse IgG, and Hoechst 33342 to stain the nuclei. EEFs were counted per well.

### *In vitro* effects of FR900098 on *B. divergens*

Erythrocytes infected with *B. divergens* were cultured in 24-well plates in triplicates for 72 h in the presence or absence of FR at different concentrations as described above. The parasitemia at time point zero was 1% in each well. The culture medium was changed daily, and parasitemia was monitored by counting Giemsa-stained blood films. From each blood film three different areas were counted. Parasitemia is expressed as the average of infected cells (from triplicates) per 100 cells.

### Assessment of the *in vivo* activity of FR900098 on *P. berghei* liver stage development

Five C57BL/6 mice were infected with 10,000 sporozoites and treated via i.p. injection with 250 mg/kg FR at 0, 12, 24 and 36 hours p.i. 5 control mice received PBS instead of FR but were otherwise treated identical. As a control for successful treatment of infection four infected mice received 60 mg/kg primaquine given 0 and 24 h p.i.

For determination of parasite load total [69], [70] liver RNA was isolated 42 h p.i. using the RNeasy kit (Qiagen), and cDNA synthesized with the RETROScript kit (Ambion), according to the manufacturer's instructions. Real time PCR was performed using the StepOne Plus real-time PCR system and Power SYBR Green PCR Master Mix (Applied Biosystems), according to the manufacturer's instructions, using gene-specific primers for the *P. berghei* 18SrRNA (for: 5′ AAGCATTAAATAAAGCGAATACATCCTTAC 3′; rev: 5′ GGAGATTGGTTTTGACGTTTATGTG 3′) and the mouse GAPDH gene (for: 5′ CGTCCCGTAGACAAAATGGT 3′; 5′ TTGATGGCAACAATCTCCAC 3′). Real time PCR was performed in triplicates, with 1 cycle of 95°C for 15 min, followed by 40 cycles of 95°C for 15 s, 55°C for 15 s, and 60°C for 45 s. Relative copy numbers were determined with the ΔΔCt method [69].

## Supporting Information

Figure S1 **Comparison of the mevalonate and DOXP pathway for the biosynthesis of the isoprenoid precursors IPP/DMAPP.** The pathways shown are based on MetaCyc [71] and were drawn using the Pathway Tools software [72]. Numbers drawn in blue are enzyme EC numbers.
(TIF)

Figure S2 **Sequence alignment of Dxr proteins from select bacteria and plastid or apicoplast-containing organisms.** Sequences were taken from NCBI and aligned using MUSCLE at http://www.phylogeny.fr/. Residues colored in red, green and black in the *E. coli* sequence are also highly conserved in all other Dxr proteins and have been implicated in binding/interaction with the substrate DOXP and/or NADPH (see [73]). Amino acids colored brown in the *T. gondii* sequence (aa 23 and 67) correspond to the aa following the predicted cleavage site of either the signal sequence (determined with SignalP 3.0) or the apicoplast targeting sequence (taken the first aa of the *E. coli* sequence as reference point), respectively.
(TIF)

Figure S3 **Localization of** ***Pf*** **Dxr in ring stages of** ***P. falciparum.*** For details see Fig. 1B.
(TIF)

Figure S4 **Localization of** ***Pf*** **Dxr in early trophozoite stages of** ***P. falciparum.*** For details see Fig. 1B.
(TIF)

Figure S5 **Localization of** ***Pf*** **Dxr in mid-late trophozoite stages of** ***P. falciparum.*** For details see Fig. 1B.
(TIF)

Figure S6 **Localization of** ***Pf*** **Dxr in late trophozoite stages of** ***P. falciparum.*** For details see Fig. 1B.
(TIF)

Figure S7 **Localization of** ***Pf*** **Dxr in merozoites stages of** ***P. falciparum*** **right before lysis.** For details see Fig. 1B.
(TIF)

Figure S8 **1D- and 3D-structures of anti-plasmodial phosphonates.** Structures of Fos and FR (**A**) and their superimposed 3D-structures (**B**). Aligned calculated three-dimensional conformer coordinates for Fos and FR were retrieved from PubChem (http://pubchem.ncbi.nlm.nih.gov) and visualized using Chimera [74]. (**C**) Structures of the two anti-plasmodial phosphonates described in [49]. See http://www.ebi.ac.uk/chemblntd/ and also the main text for details.
(TIF)

Figure S9 **Dose-dependend inhibition of [^14^C]FR uptake by two NPP inhibitors (NPPB and furosemide).** Uptake of [^14^C]FR into infected erythrocytes in the presence of different concentrations (as indicated in the figure) of NPPB (5-Nitro-2-(3-phenylpropylamino)benzoic acid) or furosemide, respectively, was determined in triplicates (see Materials & Methods). The counts were normalized to the uptake of [^14^C]FR into infected erythrocytes in the absence of inhibitor (100%), and the respective uptake determined for non-infected erythrocytes (0%).
(TIF)

Figure S10 **Expression levels of the DOXP pathway genes in** ***P. yoelii.*** The values are based on microarray data of the rodent parasite *P. yoelii* [24] and were extracted from the *P. yoelii* gene entries (accessible via the respective cross-references from the *P. falciparum* gene entries; see Table S1) at PlasmoDB (http://www.plasmodb.org/). M values denote the relative expression level between pairs of conditions, expressed as base-2 logarithm (M = ±1 means a 2-fold difference in expression between the compared samples). **BS**: mixed erythrocytic stages when parasitemia was at 5–10%. **LS24, 40, 50**: Isolated liver stage-infected hepatocytes 24, 40 or 50 hrs, respectively, after *in vivo* infection. The data indicate that some of the genes for the DOXP pathway are even stronger expressed in liver than in blood stages (negative M values).
(TIF)

Figure S11 **Western blot analysis of rabbit anti-** ***Pf*** **Dxr antisera.** The western blot of a *T. gondii* cell lysate with pre-immune sera from two rabbits (lanes 1 & 2) and the respective hyper-immune sera after *Pf*Dxr immunization (lanes 3 & 4) is shown. It clearly shows in lane 3 a very prominent band<50 kDa. This size correlates very well with a predicted molecular weight of 48.8 kDa of the mature protein (i.e. without a cleaved bipartite apicoplast targeting sequence; see also Fig. S2). The other rabbit serum did not contain specific antibodies upon *Pf*Dxr immunization.
(TIF)

Table S1 **Enzymes of the DOXP pathway of isoprenoid biosynthesis in five Apicomplexa. **Given are the EC numbers, enzyme names and respective accession numbers in either EuPathDB (http://eupathdb.org/eupathdb) for *T. gondii, N. caninum, P. falciparum* and *T. parva*, or NCBI (http://www.ncbi.nlm.nih.gov) for *B. bovis*. Designations printed in blue mean that MassSpec data have been deposited in EuPathDB for this protein (such data are available only for *Plasmodium* and *T. gondii*), indicating that this protein is present in the respective parasite stage.
(DOC)

Table S2 **Physico-chemical properties of select compounds known to act on apicoplast targets in** ***Plasmodium*** **and/or** ***T. gondii.*** Compounds were compiled from the literature [72], [73]. L-glutamic acid and pantothenic acid as physiological NPP substrates (indicated in yellow) and five other anti-plasmodials (blue) are included for comparison (see main text for details). Corresponding data were retrieved from PubChem (http://pubchem.ncbi.nlm.nih.gov/). LogD were calculated using the service ADME Boxes (http://www.pharma-algorithms.com/webboxes/). Compounds up to hexachlorophene are sorted according to their decreasing LogD. CID, compound ID at PubChem; MW, molecular weight; XlogP3, calculated Log_10_ of the partition coefficient in octanol-water [74] (http://www.sioc-ccbg.ac.cn/software/xlogp3); LogD, Log_10_ of the apparent octanol-water partition coefficient D at various pH; TPSA, polar surface area of substance [75]. * known to enter iRBC via NPP [38], [39].
(DOC)
