# Supplementary figures and images for: Fosmidomycin Uptake into Plasmodium and Babesia-Infected Erythrocytes Is Facilitated by Parasite-Induced New Permeability Pathways
Source: PLoS One. 2011 May 4;6(5):e19334. doi: 10.1371/journal.pone.0019334 (PMC3087763; doi:10.1371/journal.pone.0019334)

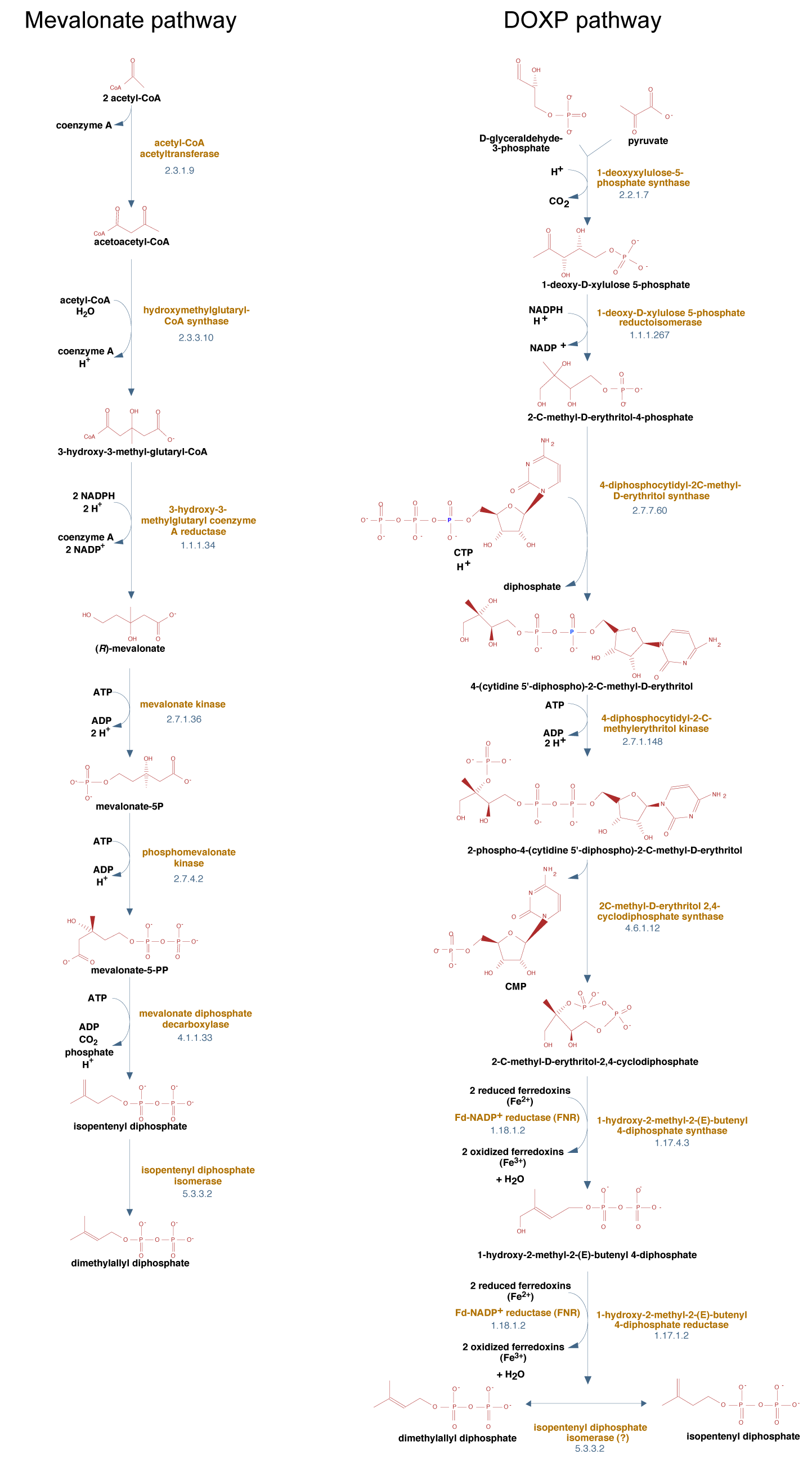

Supplement: Figure S1 — Comparison of the mevalonate and DOXP pathway for the biosynthesis of the isoprenoid precursors IPP/DMAPP. The pathways shown are based on MetaCyc [71] and were drawn using the Pathway Tools software [72]. Numbers drawn in blue are enzyme EC numbers. (TIF) [file pone.0019334.s001.tif]

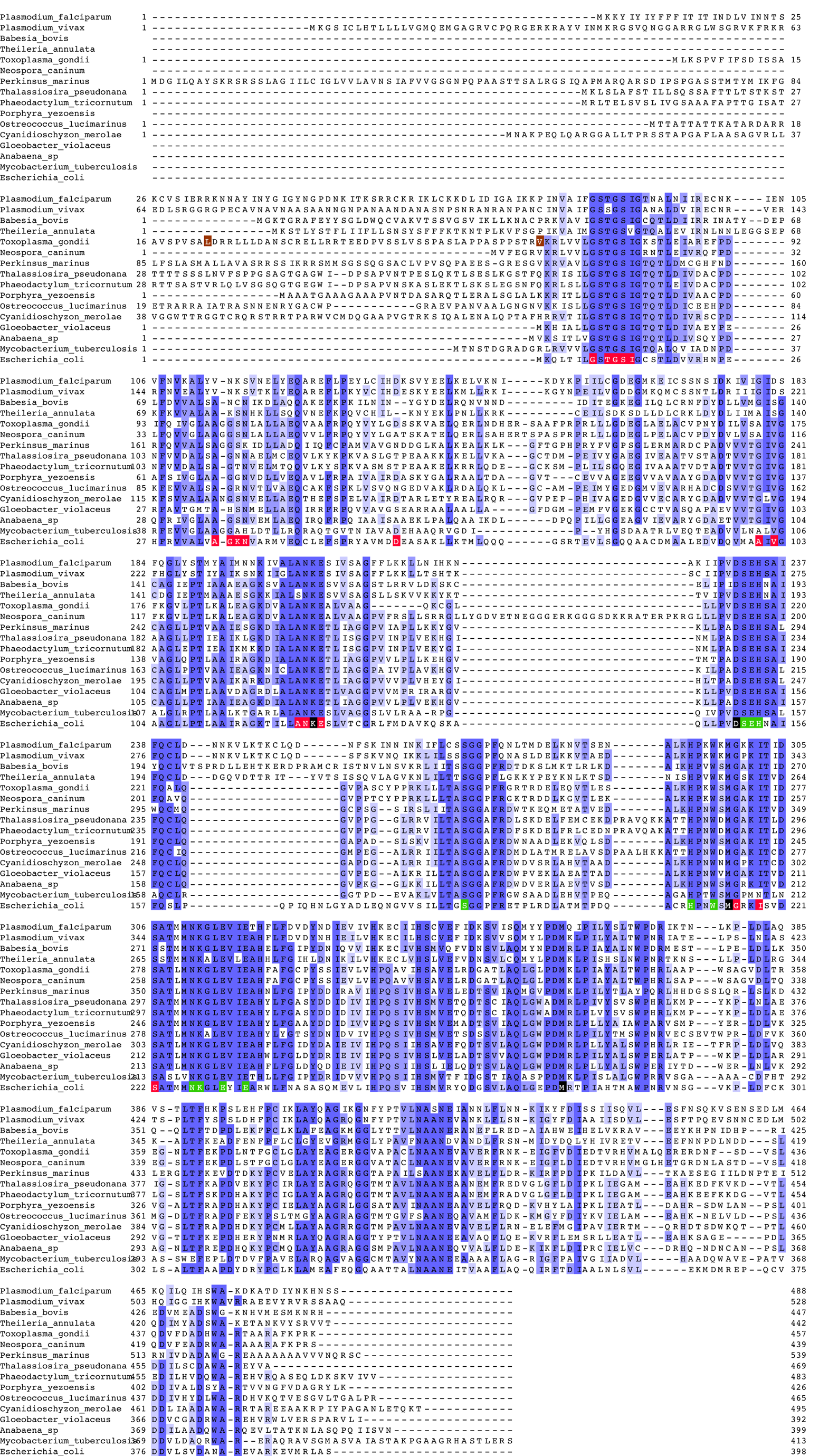

Supplement: Figure S2 — Sequence alignment of Dxr proteins from select bacteria and plastid or apicoplast-containing organisms. Sequences were taken from NCBI and aligned using MUSCLE at http://www.phylogeny.fr/. Residues colored in red, green and black in the E. coli sequence are also highly conserved in all other Dxr proteins and have been implicated in binding/interaction with the substrate DOXP and/or NADPH (see [73]). Amino acids colored brown in the T. gondii sequence (aa 23 and 67) correspond to the aa following the predicted cleavage site of either the signal sequence (determined with SignalP 3.0) or the apicoplast targeting sequence (taken the first aa of the E. coli sequence as reference point), respectively. (TIF) [file pone.0019334.s002.tif]

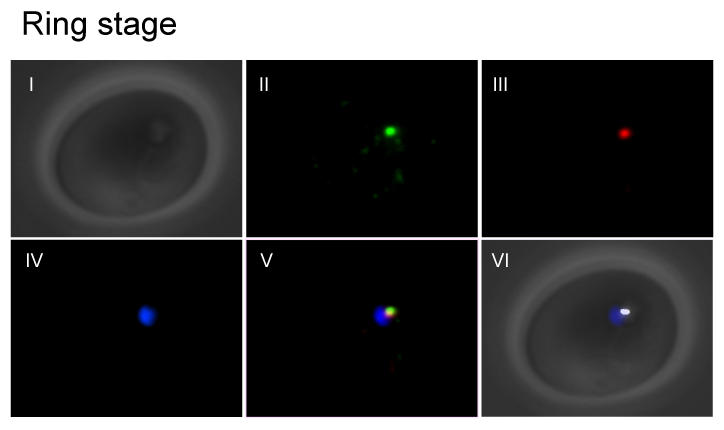

Supplement: Figure S3 — Localization of Pf Dxr in ring stages of P. falciparum. For details see Fig. 1B. (TIF) [file pone.0019334.s003.tif]

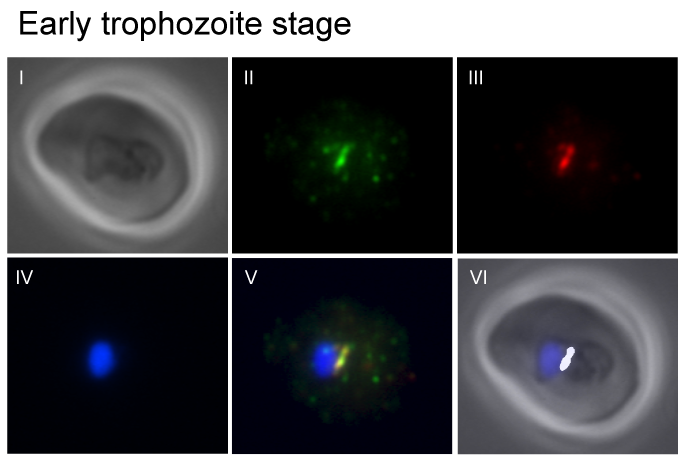

Supplement: Figure S4 — Localization of Pf Dxr in early trophozoite stages of P. falciparum. For details see Fig. 1B. (TIF) [file pone.0019334.s004.tif]

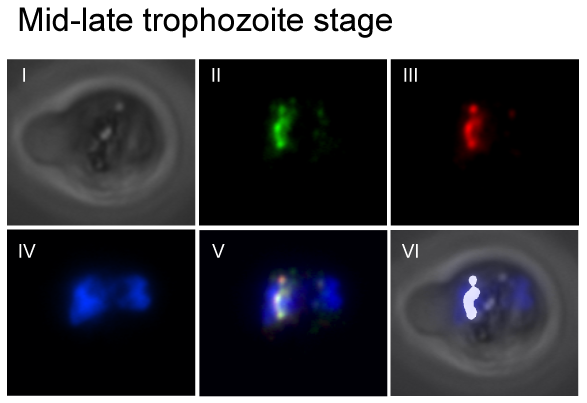

Supplement: Figure S5 — Localization of Pf Dxr in mid-late trophozoite stages of P. falciparum. For details see Fig. 1B. (TIF) [file pone.0019334.s005.tif]

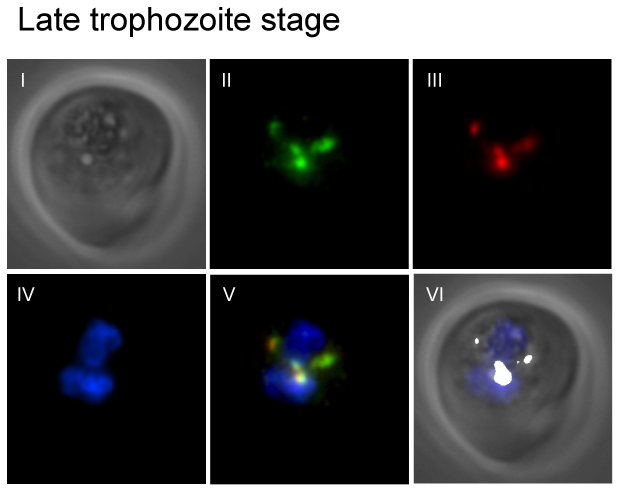

Supplement: Figure S6 — Localization of Pf Dxr in late trophozoite stages of P. falciparum. For details see Fig. 1B. (TIF) [file pone.0019334.s006.tif]

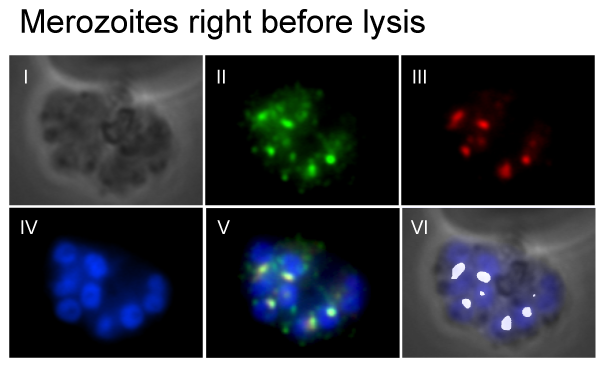

Supplement: Figure S7 — Localization of Pf Dxr in merozoites stages of P. falciparum right before lysis. For details see Fig. 1B. (TIF) [file pone.0019334.s007.tif]

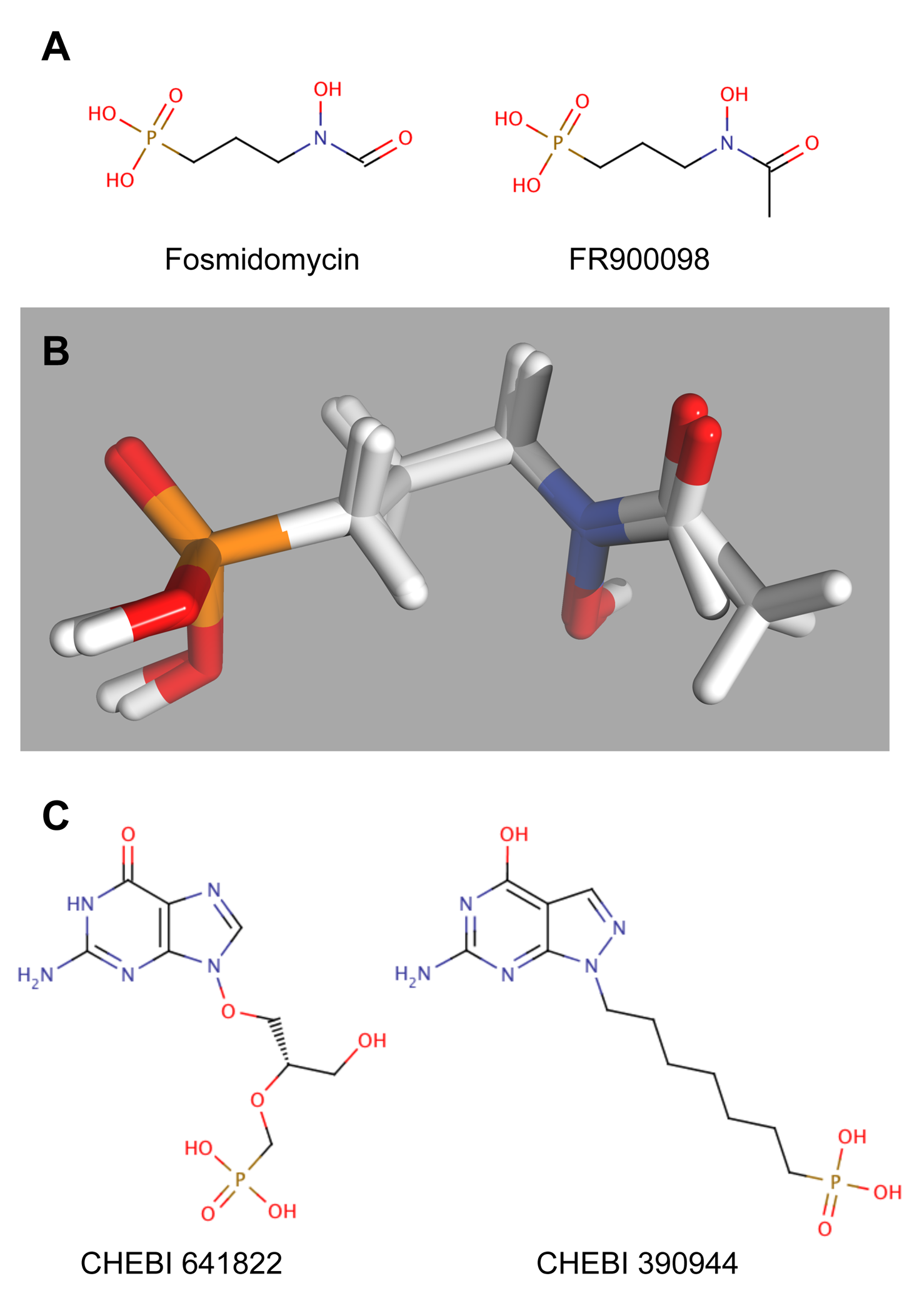

Supplement: Figure S8 — 1D- and 3D-structures of anti-plasmodial phosphonates. Structures of Fos and FR (A) and their superimposed 3D-structures (B). Aligned calculated three-dimensional conformer coordinates for Fos and FR were retrieved from PubChem (http://pubchem.ncbi.nlm.nih.gov) and visualized using Chimera [74]. (C) Structures of the two anti-plasmodial phosphonates described in [49]. See http://www.ebi.ac.uk/chemblntd/ and also the main text for details. (TIF) [file pone.0019334.s008.tif]

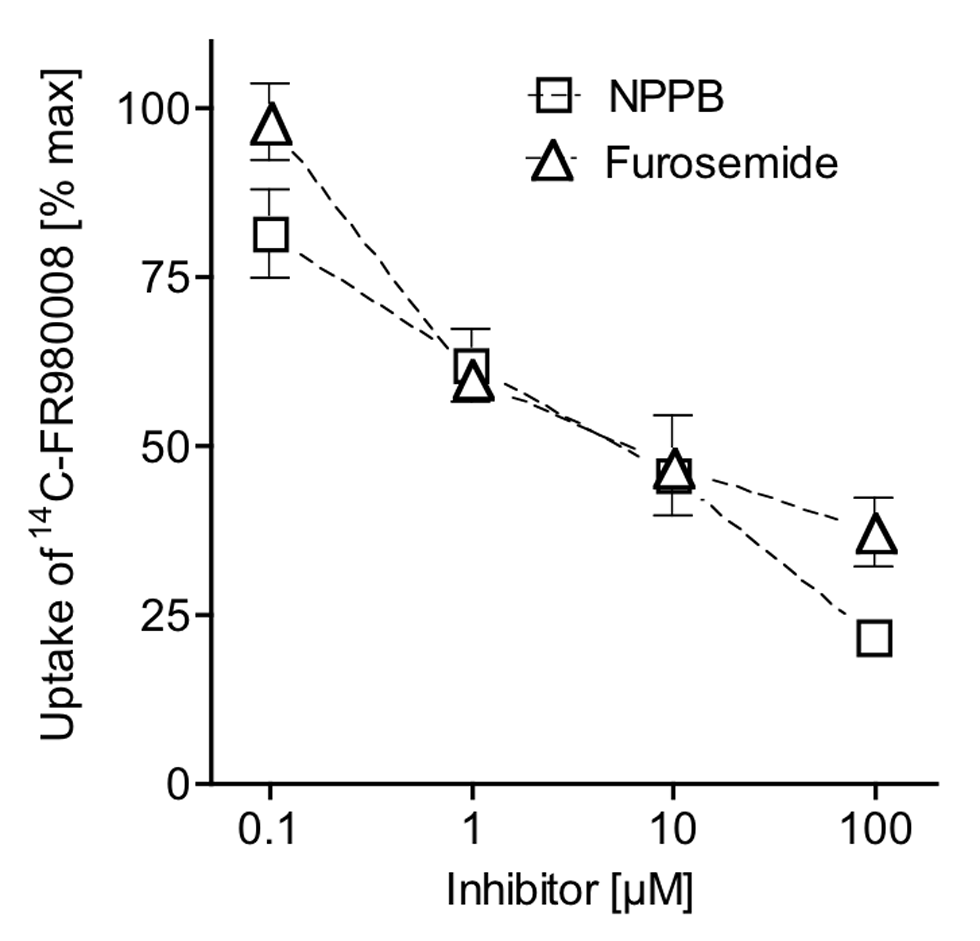

Supplement: Figure S9 — Dose-dependend inhibition of [14C]FR uptake by two NPP inhibitors (NPPB and furosemide). Uptake of [14C]FR into infected erythrocytes in the presence of different concentrations (as indicated in the figure) of NPPB (5-Nitro-2-(3-phenylpropylamino)benzoic acid) or furosemide, respectively, was determined in triplicates (see Materials & Methods). The counts were normalized to the uptake of [14C]FR into infected erythrocytes in the absence of inhibitor (100%), and the respective uptake determined for non-infected erythrocytes (0%). (TIF) [file pone.0019334.s009.tif]

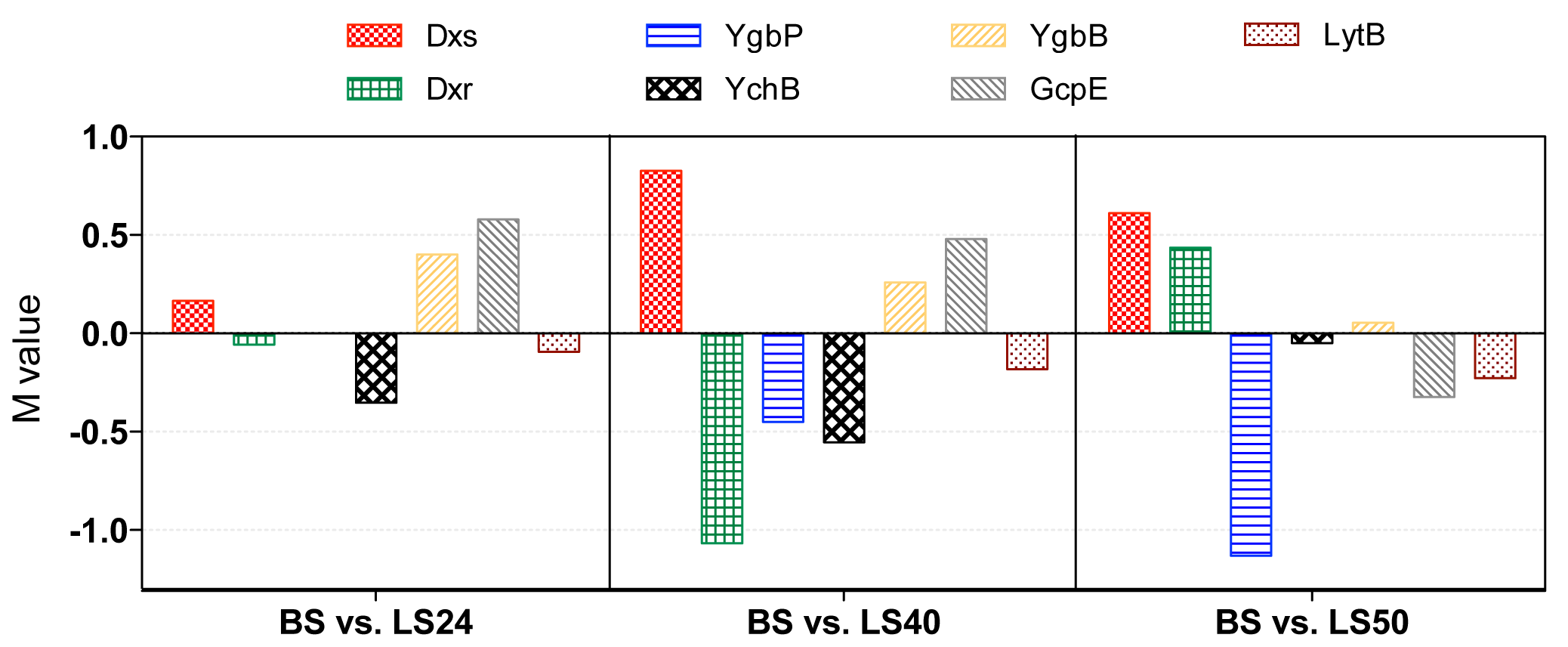

Supplement: Figure S10 — Expression levels of the DOXP pathway genes in P. yoelii. The values are based on microarray data of the rodent parasite P. yoelii [24] and were extracted from the P. yoelii gene entries (accessible via the respective cross-references from the P. falciparum gene entries; see Table S1) at PlasmoDB (http://www.plasmodb.org/). M values denote the relative expression level between pairs of conditions, expressed as base-2 logarithm (M = ±1 means a 2-fold difference in expression between the compared samples). BS: mixed erythrocytic stages when parasitemia was at 5–10%. LS24, 40, 50: Isolated liver stage-infected hepatocytes 24, 40 or 50 hrs, respectively, after in vivo infection. The data indicate that some of the genes for the DOXP pathway are even stronger expressed in liver than in blood stages (negative M values). (TIF) [file pone.0019334.s010.tif]

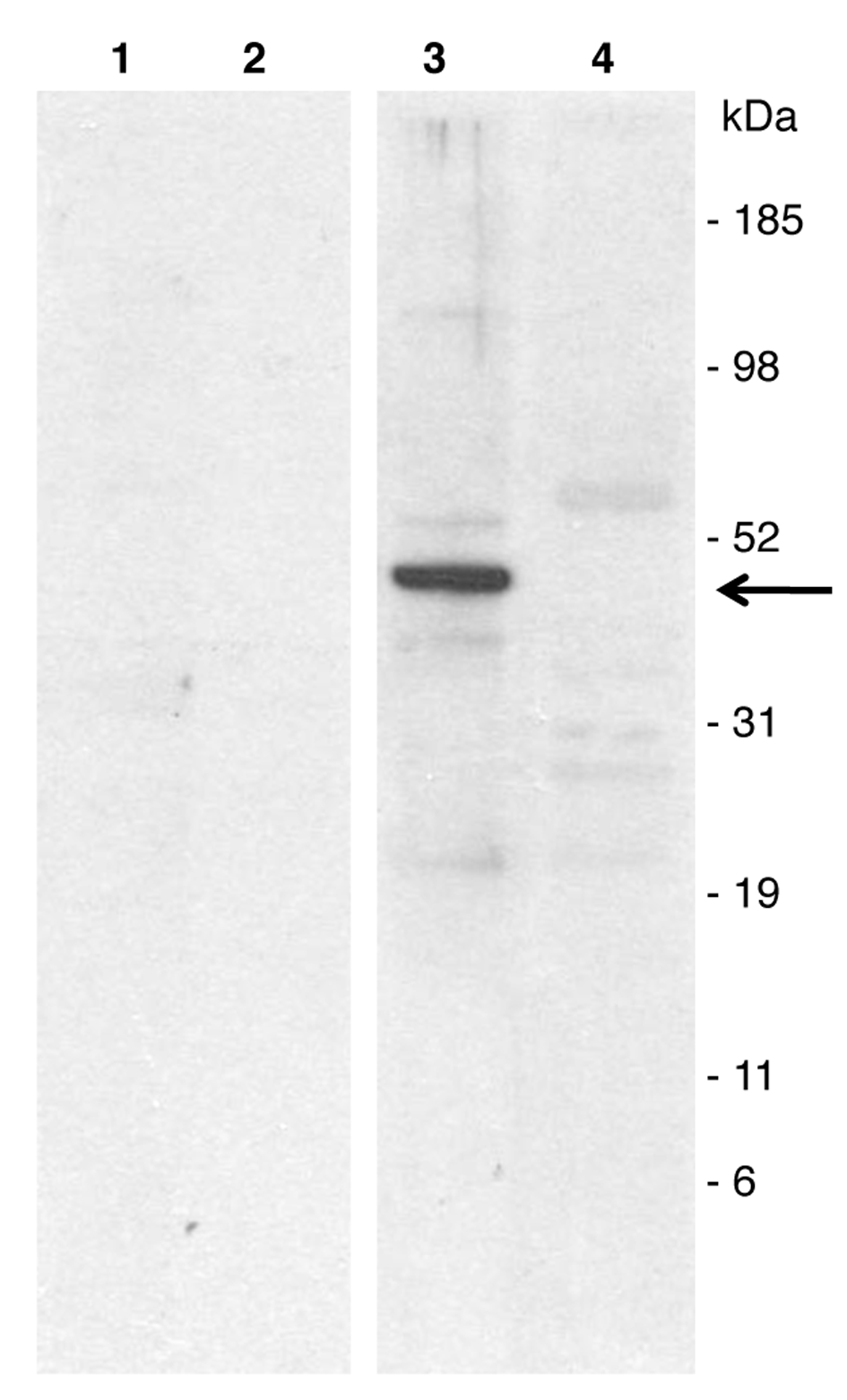

Supplement: Figure S11 — Western blot analysis of rabbit anti- Pf Dxr antisera. The western blot of a T. gondii cell lysate with pre-immune sera from two rabbits (lanes 1 & 2) and the respective hyper-immune sera after PfDxr immunization (lanes 3 & 4) is shown. It clearly shows in lane 3 a very prominent band<50 kDa. This size correlates very well with a predicted molecular weight of 48.8 kDa of the mature protein (i.e. without a cleaved bipartite apicoplast targeting sequence; see also Fig. S2). The other rabbit serum did not contain specific antibodies upon PfDxr immunization. (TIF) [file pone.0019334.s011.tif]
