# Supplementary material for: Fosmidomycin Uptake into Plasmodium and Babesia-Infected Erythrocytes Is Facilitated by Parasite-Induced New Permeability Pathways
Source: PLoS One. 2011 May 4;6(5):e19334. doi: 10.1371/journal.pone.0019334 (PMC3087763; doi:10.1371/journal.pone.0019334)
Supplement: Table S1 — Enzymes of the DOXP pathway of isoprenoid biosynthesis in five Apicomplexa. Given are the EC numbers, enzyme names and respective accession numbers in either EuPathDB (http://eupathdb.org/eupathdb) for T. gondii, N. caninum, P. falciparum and T. parva, or NCBI (http://www.ncbi.nlm.nih.gov) for B. bovis. Designations printed in blue mean that MassSpec data have been deposited in EuPathDB for this protein (such data are available only for Plasmodium and T. gondii), indicating that this protein is present in the respective parasite stage. (DOC) [file pone.0019334.s012.doc]

**Table S1**

**Enzymes of the DOXP pathway of isoprenoid biosynthesis in five Apicomplexa**

| **EC number** | **Enzyme name (Abbr.)** | ***T. gondii*** | ***N. caninum*** | ***P. falciparum*** | ***T. parva*** | ***B. bovis*** |
| --- | --- | --- | --- | --- | --- | --- |
| 2.2.1.7 | | 1-deoxy-D-xylulose-5-phosphate synthase (Dxs) | | --- | | TGME49_008820 | NCLIV_003330 | [**PF13_0207**](http://plasmodb.org/plasmodb/servlet/sv?page=gene&source_id=MAL13P1.186) | [TP01_0516](http://www.tigr.org/tigr-scripts/euk_manatee/shared/ORF_infopage.cgi?db=tpa1&orf=TP01_0516) | [BBOV_III002600A](http://www.ncbi.nlm.nih.gov/entrez/viewer.fcgi?db=nuccore&id=156087972) |
| 1.1.1.267 | | 1-deoxy-D-xylulose-5-phosphate reductoisomerase (IspC, Dxr) | | --- | | TGME49_014850 | NCLIV_051900 | [PF14_0641](http://plasmodb.org/plasmodb/servlet/sv?page=gene&source_id=PF14_0641) | [TP02_0073](http://www.tigr.org/tigr-scripts/euk_manatee/shared/ORF_infopage.cgi?db=tpa1&orf=TP02_0073) | [BBOV_III010740A](http://www.ncbi.nlm.nih.gov/entrez/viewer.fcgi?db=nuccore&id=156089574) |
| 2.7.7.60 | | 2-C-methyl-D-erythritol 4-phosphate cytidylyltransferase (IspD, YgbP) | | --- | | TGME49_106260 | NCLIV_044490 | [PFA0340w](http://www.plasmodb.org/plasmo/showRecord.do?name=GeneRecordClasses.GeneRecordClass&project_id=&primary_key=PFA0340w) | [TP03_0057](http://www.tigr.org/tigr-scripts/euk_manatee/shared/ORF_infopage.cgi?db=tpa1&orf=TP03_0057) | [BBOV_I003560](http://www.ncbi.nlm.nih.gov/entrez/viewer.fcgi?val=XM_001608956.1)A |
| 2.7.1.148 | 4-diphosphocytidyl-2c-methyl-D-erythritol kinase (IspE, YchB) | TGME49_106550 | NCLIV_044740 | [PFE0150c](http://www.plasmodb.org/plasmodb/servlet/sv?page=gene&source_id=PFE0150c) | [TP02_0681](http://www.ncbi.nlm.nih.gov/entrez/query.fcgi?cmd=Retrieve&db=Protein&list_uids=71031210&dopt=GenPept) | [BBOV_II007070A](http://www.ncbi.nlm.nih.gov/entrez/viewer.fcgi?db=nuccore&id=156085630) |
| 4.6.1.12 | | 2-C-methyl-D-erythritol 2,4-cyclo-diphosphate synthase (IspF, YgbB) | | --- | | **TGME49_055690** | NCLIV_029230 | [**PFB0420w**](http://www.plasmodb.org/plasmodb/servlet/sv?page=gene&source_id=PFB0420w) | [TP03_0365](http://www.tigr.org/tigr-scripts/euk_manatee/shared/ORF_infopage.cgi?db=tpa1&orf=TP03_0365) | [BBOV_IV002810A](http://www.ncbi.nlm.nih.gov/entrez/viewer.fcgi?db=nuccore&id=156083924) |
| 1.17.4.3 | | 4-hydroxy-3-methylbut-2-en-1-yl diphosphate synthase (IspG, GcpE) | | --- | | **TGME49_062430** | NCLIV_025400 | [PF10_0221](http://www.plasmodb.org/plasmodb/servlet/sv?page=gene&source_id=PF10_0221) | [TP02_0667](http://www.tigr.org/tigr-scripts/euk_manatee/shared/ORF_infopage.cgi?db=tpa1&orf=TP02_0667) | [BBOV_II006930A](http://www.ncbi.nlm.nih.gov/entrez/viewer.fcgi?db=nuccore&id=156085602) |
| 1.17.1.2 | | 4-hydroxy-3-methylbut-2-en-1-yl diphosphate reductase (IspH, LytB) | | --- | | **TGME49_027420** | NCLIV_045740 | [**PFA0225w**](http://www.plasmodb.org/plasmodb/servlet/sv?page=gene&source_id=MAL1P1.35) | [TP03_0674](http://www.tigr.org/tigr-scripts/euk_manatee/shared/ORF_infopage.cgi?db=tpa1&orf=TP03_0674) | [BBOV_III001660A](http://www.ncbi.nlm.nih.gov/entrez/viewer.fcgi?db=nuccore&id=156087788) |
