# Supplementary material for: Fosmidomycin Uptake into Plasmodium and Babesia-Infected Erythrocytes Is Facilitated by Parasite-Induced New Permeability Pathways
Source: PLoS One. 2011 May 4;6(5):e19334. doi: 10.1371/journal.pone.0019334 (PMC3087763; doi:10.1371/journal.pone.0019334)
Supplement: Table S2 — Physico-chemical properties of select compounds known to act on apicoplast targets in Plasmodium and/or T. gondii. Compounds were compiled from the literature [72], [73]. L-glutamic acid and pantothenic acid as physiological NPP substrates (indicated in yellow) and five other anti-plasmodials (blue) are included for comparison (see main text for details). Corresponding data were retrieved from PubChem (http://pubchem.ncbi.nlm.nih.gov/). LogD were calculated using the service ADME Boxes (http://www.pharma-algorithms.com/webboxes/). Compounds up to hexachlorophene are sorted according to their decreasing LogD. CID, compound ID at PubChem; MW, molecular weight; XlogP3, calculated Log10 of the partition coefficient in octanol-water [74] (http://www.sioc-ccbg.ac.cn/software/xlogp3); LogD, Log10 of the apparent octanol-water partition coefficient D at various pH; TPSA, polar surface area of substance [75]. * known to enter iRBC via NPP [38], [39]. (DOC) [file pone.0019334.s013.doc]

**Table S2**

**Physico-chemical properties of select compounds known to act on apicoplast targets in *Plasmodium* and/or *T. gondii***

| **CID** | **Name** | **MW** | **XLogP3** | **TPSA (Å^2^)** | **LogD at pH** | | | | |
| --- | --- | --- | --- | --- | --- | --- | --- | --- | --- |
|  |  |  |  |  | **1.7** | **4.6** | **6.5** | **7.4** | **8** |
| 572 | Fosmidomycin | 183.10 | -2.2 | 98.1 | -1.87 | -3.82 | -5.69 | -6.41 | -6.67 |
| 162204 | FR900098 | 197.13 | -2.2 | 98.1 | -1.58 | -3.53 | -5.39 | -6.11 | -6.38 |
| 2764 | Ciprofloxacin | 331.34 | -1.1 | 72.9 | -6.62 | -4.50 | -3.14 | -3.03 | -3.06 |
| 5359245 | Tetracycline | 444.43 | -1.3 | 182 | -3.49 | -2.05 | -2.03 | -2.03 | -2.05 |
| 5281011 | Doxycycline | 444.43 | 0 | 182 | -2.85 | -1.40 | -1.38 | -1.39 | -1.41 |
| 86315 | Clodinafop | 311.69 | 3.4 | 68.7 | 0.72 | 1.30 | -0.43 | -1.00 | -1.15 |
| 445629 | Thiolactomycin | 210.29 | 3.1 | 37.3 | 2.86 | 1.99 | 0.17 | -0.62 | -0.99 |
| 4421 | Nalidixic acid | 232.24 | 1.4 | 70.5 | -1.43 | 0.68 | 0.22 | -0.55 | -1.13 |
| 55185 | Azithromycin | 748.98 | 4.0 | 180 | -3.02 | -2.76 | -1.21 | -0.32 | 0.27 |
| 38687 | Diclofop | 327.16 | 5.0 | 55.8 | 4.08 | 2.58 | 0.77 | 0.20 | 0.06 |
| 6440717 | Spiramycin | 843.05 | 2.1 | 195 | -3.23 | -2.28 | -0.44 | 0.45 | 0.99 |
| 8233 | Erythromycin | 733.93 | 2.7 | 194 | -1.23 | -1.18 | -0.19 | 0.65 | 1.18 |
| 5959 | Chloramphenicol | 323.13 | 1.1 | 113 | 0.82 | 0.82 | 0.82 | 0.82 | 0.82 |
| 2020 | Actinonin | 385.5 | 1.5 | 119 | 0.98 | 0.98 | 0.97 | 0.97 | 0.95 |
| 29029 | Clindamycin | 424.98 | 2.2 | 102 | -1.53 | -1.23 | 0.35 | 1.10 | 1.40 |
| 5381226 | Rifampicin | 822.94 | 4.0 | 217 | -1.28 | -1.10 | 0.34 | 1.14 | 1.54 |
| 5564 | Triclosan | 289.54 | 5.0 | 29.5 | 4.63 | 4.63 | 4.62 | 4.53 | 4.33 |
| 3598 | Hexachlorophene | 406.90 | 7.5 | 40.5 | 7.21 | 7.20 | 6.73 | 5.98 | 5.40 |
| 611 | L-Glutamic acid | 147.13 | -3.7 | 101 | -5.14 | -4.75 | -4.74 | -4.74 | -4.74 |
| 6613 | Pantothenic acid | 219.23 | -1.1 | 107 | -0.91 | -1.45 | -3.20 | -4.05 | -4.53 |
| 6135 | Primaquine | 455.34 | -4.23 | 216 | -3.14 | -2.84 | -1.23 | -0.34 | 0.26 |
|  | T16* | 422.73 | 9.5 | 64.2 | 9.54 | 9.54 | 9.54 | 9.54 | 9.54 |
| 4735 | Pentamidine* | 340.42 | 2.6 | 118 | -2.56 | -2.55 | -2.09 | -1.34 | -0.76 |
|  | CHEBI 390944 | 329.29 | -0.11 | 147.4 | -1.13 | -1.60 | -3.44 | -4.17 | -4.43 |
|  | CHEBI 641822 | 335.21 | -2.33 | 185.8 | -5.66 | -5.54 | -6.93 | -7.62 | -7.86 |
